# Five Sectional Conferences in October

**Published:** 1916-10

**Authors:** 


### Five Sectional Conferences in October.

Anti-tuberculosis workers almost anywhere in the United States will have an opportunity to attend a sectional conference reasonably near at hand during the month of October, since five of these group meetings will be held in various parts of the country.

SOUTHERN CONFERENCE, OCTOBER 30 AND 31.

The program for the Southern conference has been arranged with particular reference to the needs of the Southern States. It will deal very largely with problems of that section of the country.

There will be a medical session on the morning of October 30, with two papers on the "Infectiousness of Tuberculosis" and "The Health Officer in the Tuberculosis Campaign." Each of these topics will be presented by an expert. On Monday afternoon there will be a general session with two round-table discussions following this session, on "Campaigning in Country Communities," led by Mr. James P. Faulkner of Atlanta, and "What Southern Cities are Doing in Tuberculosis Work," led by Mr. J. P. Kranz of Memphis.

The evening session will be of a popular nature. There will be three addresses by the following speakers: Miss Kate Gordon, Dr. Charles J. Hatfield, executive secretary of the National Association, and Dr. Oscar Dowling. The local committee of Jackson have arranged for special music, health plays and motion pictures in connection with this meeting.

A session of unusual interest will be held on the morning of October 31, at which time Mr. J. P. Faulkner of Atlanta will demonstrate with an actual group of children from the Jackson schools how to teach school children about tuberculosis. Mr. Faulkner has had wide experience and especial success in this particular line.

